# Effect of Honey (*Apis dorsata* [Hymenoptera: Apidae]) on Larval Growth and Silk Cocoon Yield of *Bombyx mori* (Lepidoptera: Bombycidae)

**DOI:** 10.1093/jisesa/iez108

**Published:** 2019-11-25

**Authors:** Muhammad Farooq Bhatti, Azizullah, Naila Shahzadi, Hafiz Muhammad Tahir, Shaukat Ali, Muhammad Tariq Zahid, Rizwan Khurshid

**Affiliations:** 1 Sericulture Wing, Forest Department, Ravi Road, Lahore, Pakistan; 2 Department of Zoology, Government College University, Lahore, Pakistan

**Keywords:** sericulture, *Morus*, silkworm, silk cocoon, supplementation

## Abstract

The present study was carried out to determine the influence of 2% aqueous honey (*Apis dorsata* Fabricius, 1793 [Hymenoptera: Apidae]) on larval growth and silk cocoon yield of fifth-instar larvae of the silkworm, *Bombyx mori* (Linnaeus, 1758) (Lepidoptera: Bombycidae). The larvae of silkworms (*Chinese HUAKAND2*) were divided into a control and an experimental groups (*n* = 20 in each group). Control group was fed with plain mulberry leaves throughout the fifth instar, whereas the experimental group was offered mulberry leaves dipped in 2% aqueous solution of honey every other day for 4 d (days 1, 3, 5, and 7). On the other days (days 2, 4, 6, and 8), plain mulberry leaves were offered to larvae. Results showed that the average weight gain in larvae of the experimental group was 348.23 and 204.54% in case of the control group. Uneaten mulberry leaves were weighed; the control group left 34.05% of their leaves and the treated group 28.54%. The cocoon formation in the honey-treated larvae was more uniform in shape than the control group. Furthermore, honey-treated larvae began to form cocoons 7.8 ± 0.23 h earlier than the control group. We also recorded an increase of 15.34% in average weight of cocoons of the experimental group when compared with the control. Average shell percentage of fresh silk cocoons of the control and experimental groups was 20.5 and 23.5%, respectively. It is concluded from the study that 2% aqueous honey has positive impact on the larval growth and cocoon yield of *B. mori*.

The silkworm, *Bombyx mori* L., is a holometabolous insect, which is reared solely on mulberry leaves (*Morus* spp.). One of the most important characteristics of the silkworm is its ability to convert plant protein into silk protein (Ude et al. 2014). It has four molts in its lifecycle in which third and fourth molt consumes more food than the first and second molt (Hussain et al. 2011). The total quantity of leaf required to raise a unit of 20,000 larvae is 400–450 kg. Along with economic and agricultural importance, it is perhaps the best model organism especially for biochemical, molecular genetics, and genomic studies (Goldsmith et al. 2005, Reumer et al. 2008). Furthermore, silk proteins have wide range of applications in medical sciences and industry (Cao et al. 2009, Jo and Um 2015, Khyade and Yamanaka 2018, Ageitos et al. 2019, Ranakoti et al. 2019).

The larval growth and production of silk by silkworm depend greatly on the nutritional composition and quality of mulberry foliage offered to the silkworms (Seidavi et al. 2005, Kantwa et al. 2006, Manjula et al. 2011). Mulberry plants play a major role in the progress of sericulture industry and in turn cocoon and silk production (Nagaraju 2002, Seidavi et al. 2005). Although the mulberry leaves are the only natural food of silkworm, however, in controlled conditions, the silkworm can be reared on artificial diets also (Saviane et al. 2014).

China is the largest silk cocoon producer in the world producing 84% of the world raw silk. India is ranked at second position contributing 14% to the world raw silk production (Popescu 2013). In Pakistan, sericulture industry is still an industry of nomads which could not be expanded on large scale due to multiple constraints including lack of trained personnel, nonavailability of pure silkworm races to produce high yield of hybrid silkworm eggs and shortage of mulberry farms, etc. (Cappellozza et al. 2002). For a long time, sericulturists have been endeavoring to enhance the yield of silk cocoon to make it commercially more viable but the explorations by the researchers are still underway. However, any cost effective intervention that increases the yield of silk cocoon with low inputs can be the most wanted step for silk cocoon–producing families across the world.

Honey contains bioactive components in the form of proteins, carbohydrates, free amino acids, trace amounts of vitamins, and metals (Falco et al. 2003, Garcia et al. 2005, Ball 2007, Moniruzzaman et al. 2014). It enhances silk productivity and quality and reduces floss output which is treated as the sericultural wastage (Thulasi and Sivaprasad 2015). Keeping in view the high nutritional and medicinal status of honey, the present study explored the possibility of including honey in the silkworm diet by analyzing its impact on the larval growth and silk cocoon production of silkworm.

## Materials and Methods

The silkworm’s eggs (*Chinese HUAKAND2*) were taken from the Sericulture Wing of Punjab Forest Department, Government of Punjab, Lahore and were reared under standard conditions of temperature (25–27°C), relative humidity (70–75%), and 16:8 (L:D) h photoperiod. Soon after hatching the larvae were placed in cardboard boxes (30 × 30 × 5 cm) at 25 ± 1°C temperature and 70–75% relative humidity. The larvae were fed with plain mulberry leaves of *Morus nigra* (hybrid variety) until the fifth instar; however, after the fourth molt, the fifth-instar larvae were divided into a control and an experimental group, each containing 20 silkworms. The larvae were enumerated and marked with blue and black ink markers on the dorsal side at the center as 1, 2, 3,…, 20 in each group to monitor their individual growth, feeding response, mobility, and increase in body weight on day to day basis. A solution of honey (98 ml water + 2 ml honey) obtained from *Apis dorsata* bees was prepared in a glass beaker and named as 2% aqueous solution of honey. The mulberry leaves were dipped in 2% aqueous honey solution for a few minutes and then air dried before presenting to larvae as a food.

**Fig. 1.**
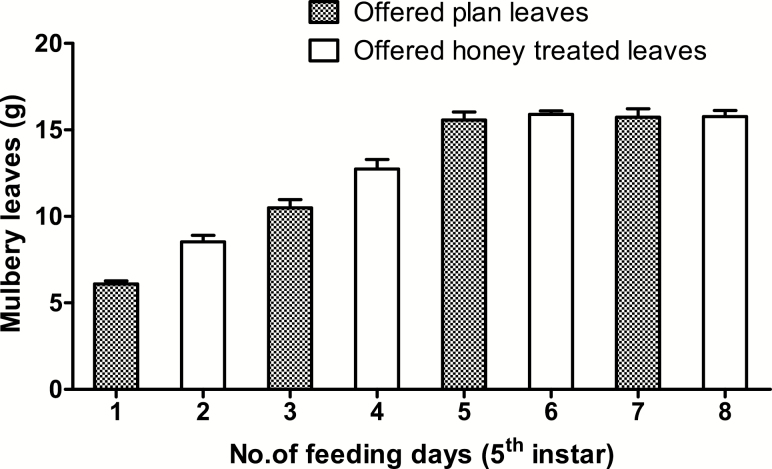
Feeding schedule of larvae of fifth instar used as experimental group.

Every 3 h in a given day, from 06:00–21:00 h, larvae were fed 6-g mulberry leaves: on average on day 1st, 8 g on day 2nd, 10 g on day 3rd, 12 g on day 4th, 15 g on day 5th, 6th, 7th, and day 8th of the fifth instar. The control group larvae were fed with plain mulberry leaves throughout the fifth instar, whereas the experimental group larvae were offered mulberry leaves dipped in 2% aqueous solution of honey on days 1, 3, 5, and 7 only. However, on the other days (days 2, 4, 6, and 8), larvae of experimental group were fed plain mulberry leaves (Fig. 1). Similarly, the uneaten mulberry leaves of control group and the experimental groups were collected before next feeding and their weights were measured and recorded regularly.

The larval weights were recorded regularly (individual and collective) just before the last feeding of the day at the time of last bed cleaning. When the larvae were mature and began to form cocoon, each cocoon box was marked with the respective number of the larva before its disappearance into the cocoon and later on the cocoons were also enumerated accordingly. Cocoon weights, shell percentage (weight of the cocoon shell ÷ weight of the whole cocoon × 100), average length, and width of cocoons of control and experimental groups were measured and recorded as it defines the quantity of silk produced by the silkworm. Repeated-measure ANOVA was used to compare the larval and cocoon weights of control and experimental groups. Independent *t*-test was used to compare the mean (±SD) weight of fresh cocoon in control and honey-treated silkworms. SPSS software was used statistical analysis.

## Results

After the first treatment (day 1), nonsignificant differences in the average weights of the control and the experimental group larvae were observed (Table 1). However, after the second treatment (day 3), a significant increase in weights of larvae was observed in the experimental group till the last and the fourth treatment on day 7. On seventh day, average weight gain of larvae in experimental groups was 348.23% of the initial average weight of day 1st when compared with 204.59% in the control group. The larvae of the experimental group gained 42.16% more weight than the larvae of the control group on the seventh day. The difference in larval weight gain of the control and the experimental group was statistically significant (*F*_1,6_ = 6.62; *P* < 0.001). After that the weights were stabilized and nonsignificant increase in weights was observed. On eighth day afternoon, the silkworms of the experimental group ripened and began to form cocoon. However, the larvae of the control group continued feeding and cocoon formation started after 7–8 h. Tables 3 and 4 show the percent increment in larval weight of control and experimental (honey-treated) silkworms larvae at different days.

**Table 1.** Comparison of mean (±SD) weight of *Bombyx mori* larvae in control and experimental groups

Days	Control group Weight gain (g)	Experimental group Weight gain (g)	Repeated-measure ANOVA
1	0.87 ± 0.41	0.84 ± 0.32	*F*_1,6_ *=* 6.62; *P* < 0.001
2	1.14 ± 0.06	1.13 ± 0.06	
3	1.45 ± 0.07	1.50 ± 0.06	
4	1.80 ± 0.09	1.99 ± 0.09	
5	2.17 ± 0.02	2.63 ± 0.12	
6	2.55 ± 0.15	3.34 ± 0.17	
7	2.66 ± 0.17	3.80 ± 0.18	

**Fig. 2.**
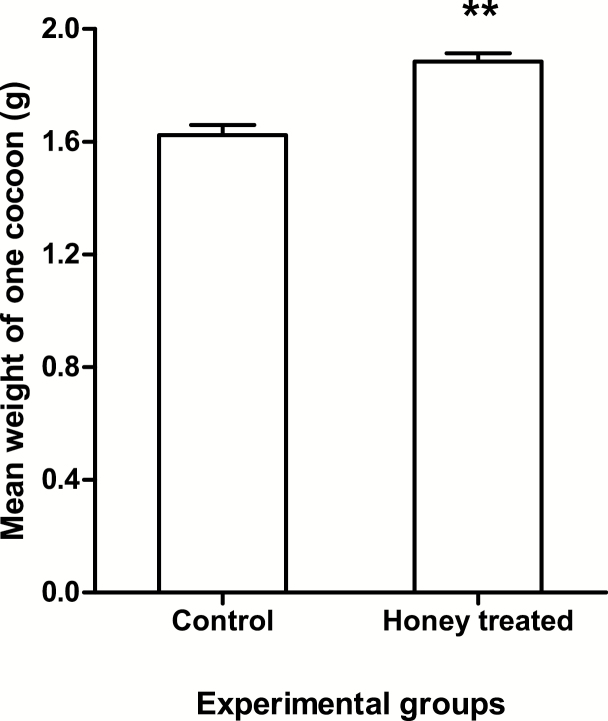
Comparison on mean (±SD) weight of fresh cocoon in control and honey-treated silkworms. A star sign on horizontal line is indicating significant difference.

Table 2 shows the comparison of consumption of food offered to the larvae and experimental groups. It is evident from Table 2 that on average experimental group larvae consumed comparatively more mulberry leaves (69.53%) than larvae of control group (65.95%). Fig. 2 shows the comparison on mean (±SD) weight of fresh cocoon in control and honey-treated silkworms. The average silk cocoon weight of the experimental group was 15.34% higher than that of the control. Similarly, the average length and width of silk cocoon of experimental group was 8.88 and 4.87% higher than control group. The average shell percentage (23.5%) was also higher than control group (20.5%). The cocoon stiffness of experimental group was felt through hand touch and found comparatively better than that of the control group. The pupae of experimental group were found to be more motile and robust than that of the control group.

**Table 2.** Comparison of food consumed by experimental and control larvae at different days of fifth-instar larvae

Days	Food offered (g)	Control		Experimental		Drying percentage
		Consumed (g)	Consumed (%)	Consumed (g)	Consumed (%)	
1	36	23.232	64.53	22.98	63.83	33%
2	48	31.086	64.76	30.9	64.375	-do-
3	60	39.096	65.16	40.2	67.00	-do-
4	72	47.262	65.64	51.54	71.58	-do-
5	90	59.838	66.48	65.58	72.86	-do-
6	90	60.24	66.93	67.14	74.60	-do-
7	90	59.916	66.57	66.9	74.33	-do-
8	90	59.202	65.78	55.32	61.46	-do-
Total	576	379.872	65.95%	400.56	69.54	-do-

**Table 3.** Measurement of weight gain (g) in larvae of in control group

No. of larvae	Nos. of days and weight gain (g)						
	1	2	3	4	5	6	7
1	0.88	1.16	1.46	1.81	2.18	2.57	2.69
2	0.88	1.14	1.45	1.78	2.15	2.53	2.63
3	0.90	1.20	1.53	1.89	2.27	2.66	2.78
4	0.87	1.13	1.43	1.77	2.13	2.50	2.61
5	0.85	1.10	1.39	1.71	2.06	2.43	2.51
6	0.87	1.13	1.44	1.78	2.15	2.53	2.64
7	0.91	1.21	1.55	1.91	2.29	2.69	2.80
8	0.88	1.17	1.5	1.86	2.24	2.64	2.76
9	0.88	1.16	1.48	1.83	2.21	2.60	2.72
10	0.87	1.13	1.44	1.78	2.15	2.53	2.62
11	0.85	1.11	1.40	1.72	2.08	2.46	2.54
12	0.88	1.15	1.46	1.80	2.17	2.56	2.66
13	0.86	1.12	1.42	1.76	2.13	2.52	2.62
14	0.86	1.12	1.41	1.73	2.08	2.44	2.53
15	0.89	1.19	1.51	1.87	2.26	2.67	2.78
16	0.91	1.21	1.56	1.95	2.34	2.76	2.88
17	0.88	1.16	1.49	1.85	2.25	2.66	2.78
18	0.88	1.15	1.46	1.80	2.17	2.56	2.67
19	0.84	1.08	1.36	1.67	2.00	2.36	2.45
20	0.85	1.1	1.38	1.70	2.05	2.42	2.51
weight gain (g)	17.49	22.92	29.12	35.97	43.36	51.09	53.18
Average weight gain (g)	0.87	1.14	1.45	1.79	2.16	2.55	2.65
Average increase from day 1 (g)	–	–	–	–	–	–	1.78
Weight gain in percent (days 1–7)	–	–	–	–	–	–	204.59%

**Table 4.** Measurement of weight gain in larvae of experimental group

	No. of days and weight gain (g)						
No. of larvae	1	2	3	4	5	6	7
1	0.84	1.13	1.5	1.98	2.62	3.33	3.79
2	0.87	1.18	1.57	2.08	2.75	3.48	3.98
3	0.83	1.11	1.47	1.94	2.57	3.27	3.723
4	0.83	1.09	1.45	1.92	2.55	3.25	3.70
5	0.81	1.07	1.41	1.86	2.48	3.16	3.57
6	0.85	1.14	1.51	2	2.65	3.36	3.83
7	0.87	1.18	1.58	2.09	2.76	3.5	3.95
8	0.87	1.18	1.57	2.08	2.76	3.49	3.97
9	0.85	1.13	1.5	1.99	2.65	3.36	3.83
10	0.82	1.09	1.44	1.89	2.51	3.18	3.62
11	0.85	1.12	1.49	1.96	2.58	3.28	3.73
12	0.88	1.2	1.6	2.11	2.78	3.51	3.99
13	0.86	1.15	1.53	2.01	2.66	3.37	3.83
14	0.88	1.19	1.59	2.09	2.76	3.49	3.97
15	0.87	1.17	1.55	2.04	2.69	3.4	3.86
16	0.84	1.11	1.46	1.92	2.54	3.32	3.77
17	0.84	1.12	1.49	1.96	2.59	3.28	3.72
18	0.83	1.07	1.42	1.87	2.49	3.17	3.62
19	0.87	1.17	1.56	2.05	2.72	3.45	3.95
20	0.84	1.1	1.45	1.92	2.55	3.24	3.8
weight gain (g)	17	22.70	30.14	39.76	52.66	66.89	76.20
Average weight gain (g)	0.85	1.13	1.50	1.98	2.63	3.34	3.81
Average increase from day 1 (g)	–	–	–	–	–	–	2.96
Weight gain in percent (days 1–7)	–	–	–	–	–	–	348.23%

## Discussion

In the present study, the impact of honey on larval growth and cocoon yield of *B. mori* was investigated. When larvae were offered mulberry leaves dipped in 2% aqueous honey solution, the silkworms initially did not show any inclination to honey-treated mulberry leaves; however, on the second and the subsequent doses, they voraciously consumed honey-treated mulberry leaves when compared with the control group. The digestive capacity of the honey-treated group also appeared to be better than that of the control group as the consumption rate and weight gain of the former were comparatively higher than the latter throughout the experiment (Rahmathulla 2012). Honey contains carbohydrates, proteins, vitamin C, vitamin B, metals, and several enzymes (Falco et al. 2003, Garcia et al. 2005, Ball 2007). Higher consumption rate of *B. mori* might be due to the variety of enzymes present in the honey that might have affected the feeding behavior and weight of treated group positively (El-Akkad et al. 2008). According to a study conducted by Sivaprasad and Thulasi (2014) on *B. mori,* honey played an important role in the growth and metabolism and its impact seemed to be concentration dependent and tissue specific.

During our study, it was observed that treated silkworms earlier as they began to form cocoon 7 to 8 h earlier than the control group. The hand touch of silk cocoons at different occasions of the spinning phase showed uniformity in hardness characteristic of silk cocoon of experimental larvae. Evidently, the spinning rate of the experimental group was more than that of the control group. The present study was carried out in the autumn season when the mulberry leaves are hard and less succulent; however, if the same study would have been carried out in the spring season when the mulberry leaves are fresh and the climate is more favorable for the growth, the larval weight and cocoon yield have been higher.

Our findings are similar to previous research studies carried out to enhance the larval growth and silk cocoon yield of *B. mori* (Nguku 2007, Wani et al. 2018, Yusoff et al. 2019). The treatment of mulberry leaves with honey is comparatively a cost effective and an easily manageable technique as most of the farmers give a dip to mulberry leaves in water to remove dust particles from the leaves. The addition of 2% *A. dorsatan* honey in water tub is neither expensive nor increase the labor cost, rather it can readily be adopted by the silkworm rearing families on account of better economic incentive and insignificant increase in cost of rearing. Moreover, this study can be a desirable intervention for the sericulturists and can play an important role to get high return from silkworm-rearing activities.
